# Fishing for nutrients in heterogeneous landscapes: modelling plant growth trade-offs in monocultures and mixed communities

**DOI:** 10.1093/aobpla/plv109

**Published:** 2015-09-14

**Authors:** Simon Antony Croft, Jonathan W. Pitchford, Angela Hodge

**Affiliations:** 1 Department of Biology, University of York, Wentworth Way, York YO10 5DD, UK; 2 York Centre for Complex Systems Analysis (YCCSA), The Ron Cooke Hub, University of York, Heslington, York YO10 5GE, UK

**Keywords:** Complexity, individual-based simulation, patchy environment, productivity, recruitment, stochastic model

## Abstract

The problem of how best to find and exploit essential resources, the quality and locations of which are unknown, is common throughout biology. For plants, the need to grow an efficient root system so as to acquire patchily distributed soil nutrients is typically complicated by competition between plants, and by the costs of maintaining the root system. Simple mechanistic models for root growth can help elucidate these complications, and here we argue that these models can be usefully informed by models initially developed for foraging fish larvae. Both plant and fish need to efficiently search a spatio-temporally variable environment using simple algorithms involving only local information, and both must perform this task against a backdrop of intra- and inter-specific competition and background mortality. Here we develop these parallels by using simple stochastic models describing the growth and efficiency of four contrasting idealized root growth strategies. We show that plants which grow identically in isolation in homogeneous substrates will typically perform very differently when grown in monocultures, in heterogeneous nutrient landscapes and in mixed-species competition. In particular, our simulations show a consistent result that plants which trade-off rapid growth in favour of a more efficient and durable root system perform better, both on average and in terms of the best performing individuals, than more rapidly growing ephemeral root systems. Moreover, when such slower growing but more efficient plants are grown in competition, the overall community productivity can exceed that of the constituent monocultures. These findings help to disentangle many of the context-dependent behaviours seen in the experimental literature, and may form a basis for future studies at the level of complex population dynamics and life history evolution.

## Abstract (precis)

Plant roots grow to acquire nutrients. Fish larvae swim to acquire planktonic food. Neither “predator” knows where its “prey” are distributed, nor what competitors also seek these resources. Both biological systems require efficient searches based on local information. Using methods originally developed for fish foraging, our theoretical study compares different modes of root growth for plants grown in isolation and in competition, and in mixed communities and monocultures. Our results may help disentangle some of the context-dependent experimental findings, for example by explaining when a plant should trade-off rapid growth in favour of a more efficient and durable root system.

## Introduction

The distribution of nutrients in soil is both spatially and temporally heterogeneous or ‘patchy’. Plants must explore this heterogeneous environment and exploit the nutrient patches they encounter to obtain the resources needed for their growth and reproduction. This exploitation is achieved via the growth of a system of roots. These roots also play important roles in anchorage and water uptake, but the uptake of nutrients is the focus of this study. In what follows, we aim to: (i) summarize the key empirical features of root growth in patchy environments; (ii) draw parallels with, and identify contrasts between, root growth and the ecological and evolutionary processes driving a seemingly rather different system, namely the foraging and growth of fish larvae, (iii) show how these similarities and contrasts can be encapsulated within mathematical, computational and statistical models. This synthesis between biological disciplines allows us to develop a modelling framework that can help to answer some important strategic questions.

Growing root systems rely on integrating local environmental information in order to efficiently exploit available resources (Robinson *et al*. 2003). Because root systems are effectively modular, and the number of modules (roots) is not fixed, growing root systems can show a high degree of flexibility or ‘plasticity’ (Hodge 2004, 2006). Moreover, roots of different plant species do not always respond in the same way to nutrient patches (Campbell *et al*. 1991; Hodge *et al*. 1998), and the same plant species grown under the same experimental conditions can show differing responses depending on the type of nutrient patch encountered (Hodge *et al*. 1999*a*, 2000*a*). This response may be further modified by the presence of competitors (Cahill *et al*. 2010; Mommer *et al*. 2012; Hodge and Fitter 2013). Consequently, general ‘rules’ of how plants will respond to their heterogeneous environment have proved hard to predict.

There is experimental evidence that individual plants respond to small-scale resource heterogeneity (defined here as heterogeneity at scales comparable to individual plant roots) through a range of mechanisms. These include increased root proliferation (Drew 1975), root production (Pregitzer *et al*. 1993; Hodge *et al*. 1999*a*, *b*), altered lateral branching (Farley and Fitter 1999; Malamy 2005) and increased ion uptake (Jackson *et al*. 1990; Robinson *et al*. 1994). Such responses vary between species and may be context-specific, for example, root growth may depend on the attributes of the nutrient patch present (i.e. size, concentration and duration; Hodge *et al*. 2000*a*, *b*, *c*).

At larger scales, and in a more ecological context, plants have evolved to grow in competition. Resource availability is known to influence plant interactions (Hodge 2004; Cahill and McNickle 2011; Hodge and Fitter 2013). It is known that heterogeneity in physical or chemical properties of soils can influence both plant diversity (Fitter 1982) and vegetation patterns (Tilman 1982) and can promote species coexistence (Berendse 1981; Fitter 1982).

Although there are clear differences between the two systems, here we argue that some of the key elements of plant root growth and nutrient acquisition have fundamental commonalities with foraging and growth of fish larvae, and that therefore there is scope for cross-fertilization between the sub-disciplines of mathematical modelling.

Two similarities are especially germane. First, like plant roots, fish larvae typically have only very temporally and spatially local information about their environment. Nor are they renowned for their intellectual capacities. While factors such as turbulence, detailed fluid mechanics, environmental heterogeneity and predator–prey interactions may all play a role (Pitchford and Brindley 2001; Pitchford *et al*. 2003), the paradigm of an essentially agnostic and unintelligent biological entity (plant root or fish larva) foraging for heterogeneous resources using only local information is identical.

The second, less immediately obvious, commonality concerns the interplay between the roles of populations (of roots from a single plant, or of offspring from a single parent fish), evolution and ‘luck’. An adult female fish will typically produce millions of eggs. Assuming equal sex ratios and constant population size and structure she needs two of these to hatch and grow to maturity over her lifetime; only a tiny minority of larvae, the ‘luckiest’, successfully reach adulthood (Pitchford *et al*. 2005). Evolution would therefore favour behaviours that increase the probability of an individual being ‘lucky’ (e.g. the ability to find, remain within and exploit an ephemeral food patch) rather than those which confer an advantage on average (e.g. faster swimming) (Pitchford *et al*. 2003). The success of a plant at below-ground resource capture, in contrast, depends on the integrated performance (and cost) of all of its constituent population of roots. However, each growing root could be thought of as an essentially independent forager seeking to exploit nutrients while subject to the possibility of mortality (root ‘turnover’). It is not immediately clear whether investing in a population of fewer more resilient roots may confer more of a benefit to the plant than a larger number of faster growing, more ephemeral, roots. Plants generally have both root ‘types’, but the balance between the two differs among species.

The mathematics of stochastic (‘random’) processes provides the unifying tool to quantify these ideas. First, stochastic models of individuals foraging in patchy environments developed for fish larvae, can be transferred to the analogous plant root system. Secondly, the impact of individual-level variability at the population scale can be addressed: the crucial ingredient here is that in non-linear stochastic systems one cannot simply multiply the average success of an individual by the population size to estimate population-level performance. Jensen's well known (to statisticians) inequality states that ‘the function of the average is not the same as the average of the function’ (see, for example, Pitchford *et al*. 2005), and therefore more mathematically rigorous methods are required.

The preceding comments allow a logical framework to be developed which applies expertise and methodologies from models of larval growth to be transferred to plants. Several authors have applied models of animal behaviour to plants (Gersani *et al*. 2001; Maina *et al*. 2002; McNickle and Brown 2014) with varying degrees of success (see Hess and de Kroon 2007; Hodge 2009; Dudley *et al*. 2013). Nevertheless, the application of animal-inspired models to plant foraging offers a useful way forward, particularly given the difficulty in studying individual root systems in the first place, let alone the more realistic case when these have evolved to grow in a complex plant community.

This study uses methods motivated by foraging fish larvae to explore the growth of plant roots in an unpredictable and heterogeneous environment at the root system scale, and to account for intra- and inter-specific competition between plants with contrasting growth strategies. Growth models employing stochastic differential equations (SDEs) provide general results about the role of randomness (Pitchford *et al*. 2005). For animal foraging, extending these to so-called non-diffusive systems (allowing for more realistic movement patterns) has been particularly useful (Sims *et al*. 2008; Preston *et al*. 2010), but there are still open problems (Pitchford 2013). Perhaps more notably in this context, SDE results derived for fish (Lv and Pitchford 2007) have been applied to plant monoculture data using Bayesian methods to identify and quantify plant root competition at a phenomenological rather than at a mechanistic level (Lv *et al*. 2008).

In Croft *et al*. (2012), an idealized 1D model of plant growth, root proliferation, resource capture and inter-plant competition was developed and shown to match SDE representations; this model was used to study the effects of spatial heterogeneity in resource distribution on the evolutionarily optimal root proliferation strategy in monocultures. Details of the model implementation, and of its practical equivalence to SDE models, are provided in Croft *et al*. (2012). A hierarchy of factors emerged, with the ‘optimal’ (in an evolutionary context) root proliferation strategy depending on resource levels and their distribution, and on the presence or absence of competition.

In the present work, the model from Croft *et al*. (2012) is firstly adapted and expanded into two spatial dimensions, and secondly extended to allow competition between several plant species. These developments, although necessarily ‘strategic’ in that they describe idealized growth and competition scenarios rather than particular species and environments, allow the trade-off between different root system growth strategies to be modelled explicitly. This allows the model to capture spatial and temporal crowding effects and plant–plant interactions, as well as more realistic resource distributions. It also allows results relating to growth in monocultures to be distinguished from the behaviour of mixed competitive communities.

Plants are modelled with different growth properties, some growing quickly at the sacrifice of the effectiveness of the root system to capture and uptake available nutrients, and others trading off speed and initial size for a root system better at capturing local resources (cf. the fish larvae modelled in Pitchford *et al*. (2003) and Preston *et al*. (2010), wherein the trade-off is between swimming faster to incur a deterministic cost in the hope of a stochastic gain in prey encounters). Spatially averaged resource densities are the same between different environmental types, but the relative levels of resource heterogeneity differ (again following the analogy of Pitchford and Brindley (2001), Pitchford *et al*. (2003), Preston *et al*. (2010)). These ecological extensions to the established idealized model provide a theoretical framework within which to ask three important strategic questions:

1. How does the growth strategy adopted by a single plant impact upon its performance in a monoculture?
2. When plant species are grown in mixed competition for resources, what is the impact on individual, population and community productivity?
3. What is the role of resource heterogeneity in the above questions?

Answers to these questions are of importance to food security and the development of efficient agriculture, and are also relevant to more general issues of ecological diversity and productivity. The methods used are necessarily idealized but can offer useful general insights and to provide focus for future theoretical and experimental work.

## Methods

### Overview

A new computational model was created within the Matlab-coding environment, building upon methodologies developed, tested and described in detail in Croft *et al*. (2012) and Croft (2013). At its core, the model allows the root systems of individual plants to grow and compete for finite resources, using probabilistic methods to allow the broad-scale properties of root system growth, and the stochastic interactions between roots and environment, to be described with a small number of parameters.

The model is summarized conceptually, below, with emphasis on the strategic modelling approach and the key biological factors: different root system growth strategies; descriptions of environmental heterogeneity and the contrasts between isolated growth, monocultures and mixed communities. Technical details of mathematical and computational implementation are available in the Technical Methodology and in Croft (2013) **[see Supporting Information—File S1]**.

The parameter values chosen in this study are given in the Technical Methodology **[see Supporting Information—File S1]**. These should be considered only relative to one another, rather than as pertaining to any particular biological system. In this sense, the total time (*T*=1) for each simulation is arbitrary. It may be helpful to think of this time scale as referring to a single growing season. The plants have intrinsic growth rates (*g*) allowing them to approach some upper size limit (*L*_max_) on this time scale, but this size limit also depends on the success of their root system in finding resources. These resources are distributed throughout the environment as a set of *n* individual point resources, which may be encountered by a growing root system. The efficiency of the root system in finding these resources is described by the root system efficiency (SDE) measured on a scale of 0 (no utilization of encountered resources) to 1 (perfect utilization). In this way, the trade-off between growing fast but potentially unreliable root systems can be contrasted with more efficient but slower growing roots. The model updates on a time scale (*dt* = 10^−4^) in the order of 1 h.

### Modelling the resource environment

The environment is defined as a square of continuous space with periodic boundaries (i.e. one edge connects to the opposite edge). The environments are sized sufficiently large so as to not inhibit growth of an isolated individual due to space limitation, and are scaled according to the number of plants being grown within a numerical simulation so that plant density (in terms of number of plants per unit area) is constant. These two measures ensure that space is not a limited resource at the population level, and facilitate comparison across all simulation scenarios.

Resources occur in the environment in a finite number of discrete locations. Each of these discrete resources is of the same quality, i.e. it confers the same relative growth benefit to a plant able to acquire it.

Across all environments, the mean resource density is kept constant. Combined with the spatial scaling detailed above, this ensures that the total quantities of resources per plant, as well as the total resource density across the entire environment, are consistent across all scenarios. This allows the role of resource heterogeneity to be addressed without ambiguity.

**Figure 1.**
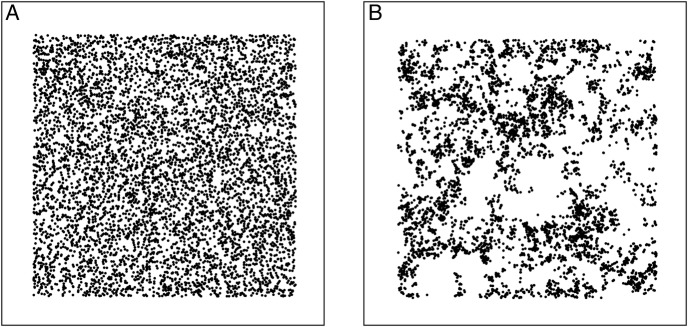
Visual representations of a ‘uniformly random’ environment (A) and ‘patchy heterogeneous’ environments (B). Each environment is comprised of 6400 individual point resources. In uniformly random environments, these are distributed according to a 2D independent uniformly random distribution. There is no structure to the patch distribution, with each patch independent from all others. In patchy heterogeneous environments, resources are distributed according to a 2D random walk. The random walk step lengths are sampled from a long-tailed Pareto distribution, and rotations sampled from a uniformly random distribution. Individual resource positions are not independent, with a patchy heterogeneous structure emerging.

Two types of probabilistic environmental heterogeneity are considered: ‘uniformly random’ and ‘patchy’. The uniformly random environments (Fig. 1A) are created by placing each discrete resource within the environment independently according to a 2D uniform random distribution. This creates a statistically homogeneous environment, with a given resource point providing no information about the relative location of any other. In contrast, the patchy environments (Fig. 1B) are created by a random walk process sampling rotations from a uniformly random distribution, and step lengths from a long-tailed Pareto distribution (Preston *et al*. 2010). This results in statistically ‘patchy’ environments, where individual resource points are likely to aggregate to form a structured distribution.

This approach assumes that nutrient resources occur as a finite number of discrete points. However, because the simulations use a large number of resource points, the overall resource distribution is essentially continuous at the scale of a plant (see Figs 1, 6, and 7) while maintaining a computationally tractable model.

### Randomizing initial plant locations

When grown in isolation, an individual plant is placed in the centre of the environment. Since the boundaries are periodic and the environments randomly generated, it does not actually matter where in the environment an isolated plant is placed (i.e. there will be no boundary effects or environmental bias by being placed centrally); the centre is chosen merely for convenience.

When a simulation is to comprise of multiple plants growing and competing simultaneously (either as a monoculture or in mixed competition), each individual is placed independently according to a 2D uniform distribution within the environment. This means that the placement of each individual is random within the environment, and that the presence/absence of competitors within an area, or the type of plant, does not affect this placement. The resulting distributions of competitors within the neighbourhood, which are statistically uniform on average, may lead to varying levels of localized grouping and competition within and across each realization of the simulations.

### Implementing root system growth

Each individual plant's root system starts as a point and expands radially (i.e. as an expanding circle) with growth at a constant rate (by area). Each individual has its own initial upper size limit, and growth ceases when the plant reaches this size. This initial upper limit can be thought of as representing possible growth due resources in the seed and/or background resource concentration, and is necessary to ‘kickstart’ the growth/resource acquisition. This initial size limit is parameterized to be equal to one-tenth of the expected final size of an individual growing in isolation with available resources.

Whenever a plant's root system expands to overlap a resource point, the plant has a chance (detailed in Growth strategies and competition section) to acquire this resource and allocate it to growth.

With the successful acquisition of each resource point, the plant experiences an instantaneous growth (i.e. a jump in size), and the upper size limit increases by an amount equal to the growth jump (i.e. growth is resource limited, and by acquiring resources this ceiling limit on size increases). The size of this jump is equal to the quality of the patch, *p*, and the individual plant's relative marginal benefit factor parameter, *mbf*. Individuals are not directly affected by competing neighbours, so root systems can overlap. Indirectly, plants growing in crowded areas, and whose root systems overlap with neighbours, risk finding themselves growing into areas depleted of resources by their competitors.

This method has been shown to successfully replicate the non-linear growth of an individual growing according to Gompertz growth functions (Purves and Law 2002; Schneider *et al*. 2006; Lv *et al*. 2008), where resource acquisition results in an increase in asymptotic limit and current growth rate (Croft *et al*. 2012; Croft 2013), as well as preserving results of competition between multiple plants (Croft 2013). It is noted that the Gompertz equations arise naturally via the Von Bertalanffy fish growth models (Lv and Pitchford 2007) which motivated this work. Simulating with linear growth and instantaneous resource depending growth as described here is significantly computationally quicker than direct implementation of Gompertz models (Croft 2013).

Note that, because this model concerns below-ground interactions, plant growth and root system growth are synonymous; one can consider above-ground growth to be reflected by below-ground growth, with above-ground effects such as shading and carbon limiting neglected (i.e. growth is purely below-ground resource limited). Root systems appear as circles representing their size, but this does not prevent the model from probabilistically accounting for finer scale structure, as detailed below.

### Growth strategies and competition

The model allows root system growth strategies involving rapid growth of ephemeral and/or sparse root systems to be distinguished from those involving slower growth and possibly more exhaustive exploitation of local surroundings. Explicitly, at any time each plant's root system has a size (area) *A* and a probability determined by its ‘RDE’ of acquiring available resources which its root system overlaps.

**Figure 2.**
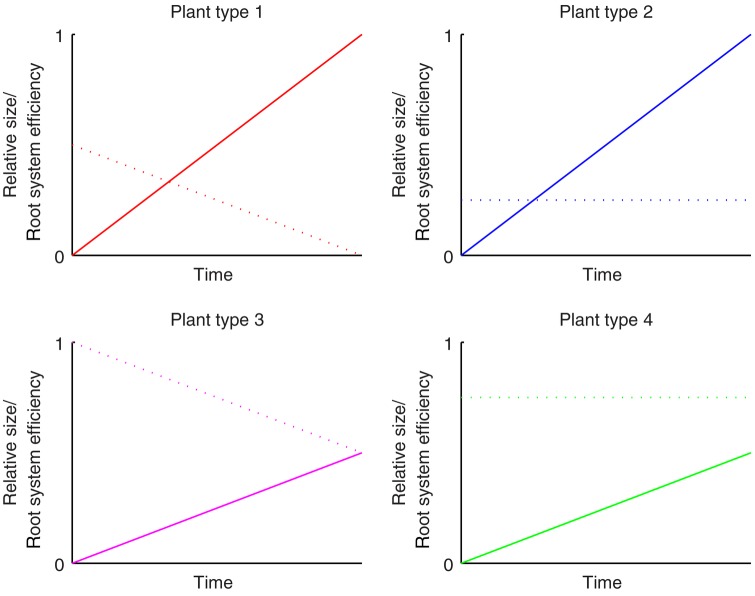
Visual representations of different growth strategies. The solid lines denote relative growth, and the dotted lines relative *RSE*. Plant types 1 and 2 experience faster growth rates at the expense of lower *RSE*; Plant types 3 and 4 instead have slower growth rates but higher *RSE*. Plant types 1 and 3 have declining *RSE*, while Plant types 2 and 4 have constant *RSE*, equal to the average *RSE* of types 1 and 3, respectively. Plant type 1 is represented by red lines, type 2 by blue lines, type 3 by magenta lines and type 4 by green lines. This colour coding will remain consistent throughout subsequent figures.

Figure 2 summarizes, schematically, the way in which these properties change with time for four contrasting idealized plant growth strategies (labelled ‘species’ for conciseness). Plants of type 1 are represented by red, type 2 by blue, type 3 by magenta and type 4 by green. For clarity, this colour scheme is maintained throughout all subsequent figures, with darker shading to indicate plants grown in uniformly random environments and lighter shading to indicate growth in patchy environments.

Strategies are defined by relative growth rates and relative abilities to acquire available resources. As well as relative levels, some plants exhibit a constant ability to obtain available resources, while others see this ability decline with time. Type 1 and 2 both grow equally quickly, and have the same average *RSE* throughout the period of simulation. However, type 2 has a root system whose *RSE* starts relatively high and then declines with time (reflecting an ephemeral root system where the ability to forage effectively diminishes as the root system becomes more diffuse) whereas type 2 has a constant *RSE* (reflecting more investment in maintenance of the root system at the expense of initial efficiency). Type 3 and 4 grow slowly (relative to type 1 and 2), but they benefit from investing in a more efficient root system (i.e. one which will statistically capture more available resource per unit area occupied) which better exploits available resources in a way which either starts high and declines with time (type 3) or remains constant with time (type 4).

Parameter values for resource quantity/quality are chosen such that, when grown in isolation in uniformly random environments, all four plant species perform equally well on average. This provides a normalized level of performance against which to measure the relative performance of the different plant species in varying conditions. By accounting for trade-offs in this way and normalizing behaviour in idealized conditions, the study retains its focus on the role of intra- and inter-specific competition, and its modulation by resource heterogeneity.

## Results

**Figure 3.**
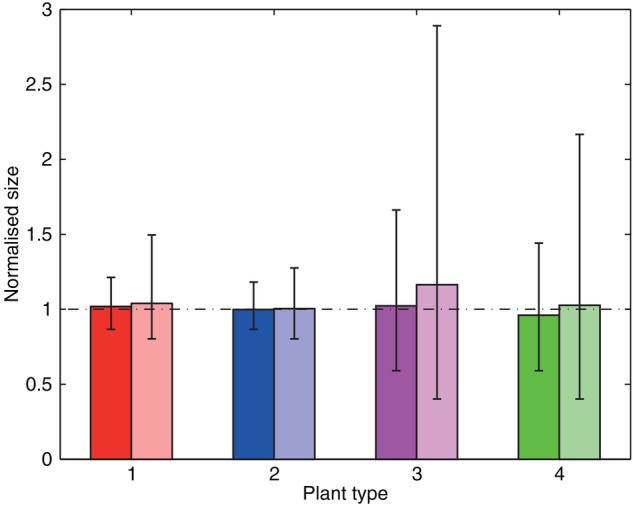
Normalized mean size of individuals grown in control conditions (i.e. in isolation) in uniformly random (darker bars) and patchy heterogeneous (lighter bars) environments. The mean size across all four plant types in the uniformly random environments is taken as the base level to which results are normalized. Results for each plant type/environment type combination show mean size for 10 000 repetitions, with vertical bars denoting 5th and 95th percentiles. Plant type 1 is represented by red bars, type 2 by blue bars, type 3 by magenta bars and type 4 by green bars.

**Figure 4.**
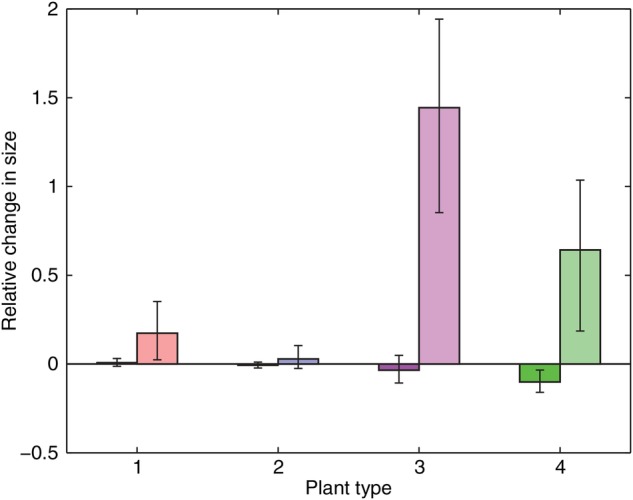
Relative change in size for each plant type when grown as a monoculture (in competition with its own kind) compared with baseline (control tests in uniformly random environments) results. Darker bars show results in uniformly random environments, with lighter bars showing results in patchy heterogeneous environments. Vertical bars denote 5th and 95th percentiles for normalized population level results across 100 repetitions of 64 plants. Plant type 1 is represented by red bars, type 2 by blue bars, type 3 by magenta bars and type 4 by green bars.

**Figure 5.**
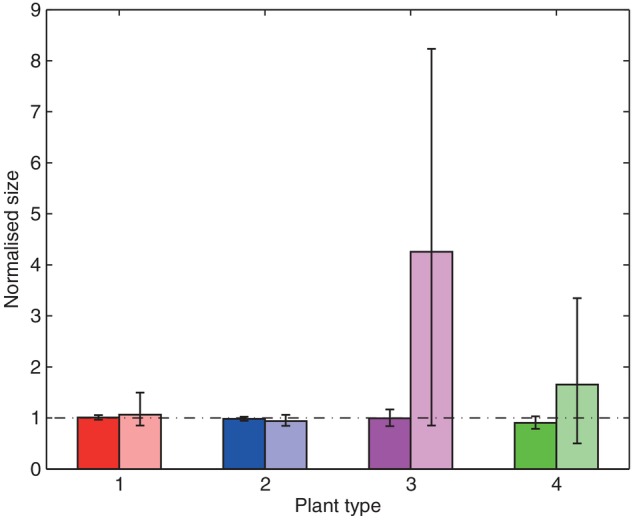
Normalized mean size of individuals when grown in mixed competition with all plants types. Results normalized against baseline (control tests in uniformly random environments) results. Darker bars show results in uniformly random environments, with lighter bars showing results in patchy heterogeneous environments. Vertical bars denote 5th and 95th percentiles for normalized plant type population level results across 1000 repetitions of 64 plants (16 of each type). Plant type 1 is represented by red bars, type 2 by blue bars, type 3 by magenta bars and type 4 by green bars.

**Figure 6.**
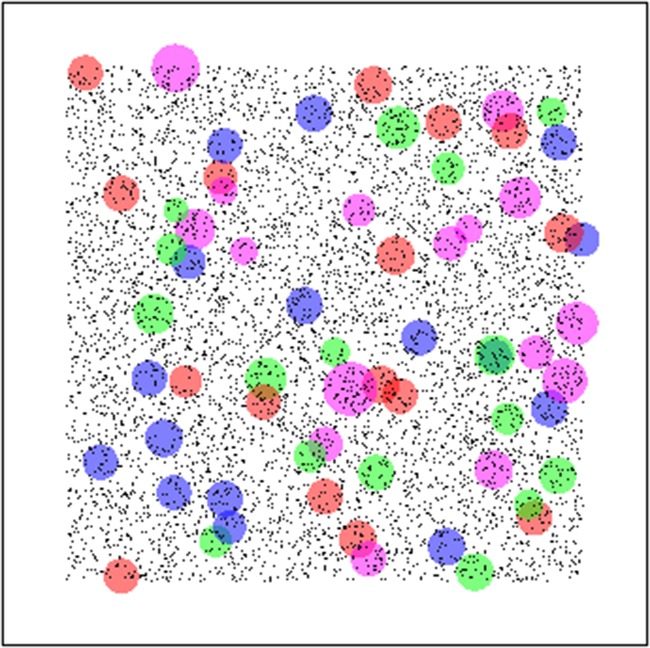
Visual representation of mixed competition experiments in a uniformly random environment. Population comprised of 64 plants (16 of each plant type) placed uniformly randomly within the environment. Environment has periodic boundaries which are not shown in this figure for clarity of distribution of individuals and their sizes. Plants of type 1 are represented by red circles, type 2 by blue circles, type 3 by magenta circles and type 4 by green circles.

**Figure 7.**
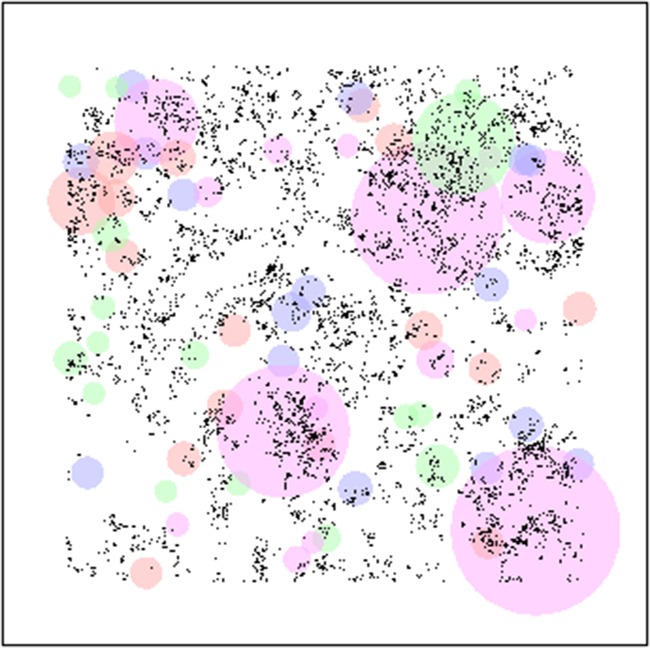
Visual representation of mixed competition experiments in a patchy heterogeneous environment. Population comprised of 64 plants (16 of each plant type) placed uniformly randomly within the environment. Environment has periodic boundaries which are not shown in this figure for clarity of distribution of individuals and their sizes. Plants of type 1 are represented by red circles, type 2 by blue circles, type 3 by magenta circles and type 4 by green circles.

The numerical implementation of the model is carried out as follows. First, in a series of ‘control’ tests, a single individual is placed in an environment and allowed to grow in the absence of competition. The results from this (Fig. 3) not only confirm that, on average each of the ‘types’ of plant under consideration performs equally well, but also illustrate where environmental heterogeneity can cause substantial variability about that average. Having established a level playing field for plants in isolation, the simulations are then extended to model the growth of several plants competing within a monoculture (Fig. 4), and finally to investigate competition and growth within a mixed community (Figs 5–7).

The different plant types were tested in control, monoculture competition and mixed competition conditions within the uniformly random and patchy environments. Throughout the results (Figs 3–5), each pair of grouped bars represent an individual species, with the darker (left hand) bars signifying growth in uniformly random environments, and the lighter (right hand) bars growth in patchy environments. The different plant types continue to be represented in figures by the same colours as in Fig. 2.

### Control tests: one plant in isolation

The results for the control tests (individuals grown in isolation) are summarized in Fig. 3. Bars show average performance across 10 000 individuals, with the 5th and 95th percentiles shown to demonstrate relative variability.

#### Uniformly random environments

As discussed in Methods, the quality of the individual resource patches (in terms of the marginal benefit to the acquiring plant) was chosen so as to best normalize performance across the four different plant types in control conditions within uniformly random environments. As such, when grown in isolation within the uniformly random environments, average performance is relatively even among the different plant types and their different growth strategies, with Plant types 3 and 4 (the slower growing plants types with higher *RSE*) showing higher relative variability.

#### Patchy environments

When the control tests are repeated within the patchy environments, average performance remains largely unchanged from the comparative results for uniformly random environments. All plant types exhibit little change in average performance, but Plant types 3 and 4 experience a significant increase in variability.

### Monoculture tests: intraspecific competition

Figure 4 shows the relative change in performance for each of the four plant types when grown as a monoculture in competition. Relative performance is gauged against the baseline normalized performance for individuals grown in control conditions in uniformly random environments (Fig. 3). Bars show average performance across 100 populations of 64 plants, with the 5th and 95th percentiles shown to demonstrate relative variability.

#### Uniformly random environments

None of the plant species exhibit any important change in average performance when grown within competitive monocultures in the uniformly random environments. Plant types 1 and 2 (the faster growing plants types with lower *RSE*) also exhibit very little variability, but Plant types 3 and 4 (the slower growing plants types with higher *RSE*) demonstrate slightly higher variability, with type 4 (constant *RSE*) showing a small reduction in average performance.

#### Patchy environments

The introduction of competition within monocultures in patchy heterogeneous environments sees a significant shift in the relative performance across the different plant types. Of the two faster growing plant types with lower *RSE*, Plant type 1 (decreasing *RSE*) demonstrated a small increase in performance while Plant type 2 (constant *RSE*) experienced little difference compared with the control tests. In contrast, the slower growing plant types with higher *RSE* exhibited significant gains in performance when grown as monocultures compared with in control conditions. Plant type 3 (declining *RSE*) experienced a much larger gain than type 4 (constant *RSE*). Variability of results also increased markedly, especially in Plant types 3 and 4.

### Mixed competition: community-level productivity

The results for when all four plant types are grown simultaneously in mixed competition are summarized in Fig. 5. Bars show the average performance across 1000 sub-populations of 16 plants, with the 5th and 95th percentiles shown to demonstrate relative variability.

#### Uniformly random environments

When grown in mixed competition within uniformly random environments, none of the four plant species demonstrated any significant difference in performance from the control baseline result. Figure 6 shows a visualization of one of the simulation runs.

#### Patchy environments

Growing all four plant types together in mixed competition within patchy environments resulted in significant gains for Plant type 3. Plant type 4 had little change from performance as a monoculture, but still outperformed the faster growing plants types with lower *RSE* (types 1 and 2) which demonstrated little difference in performance from previous numerical simulations. Plant types 3 and 4 (the slower growing plants types with higher *RSE*) demonstrate large variability. Figure 7 shows a visualization of one of the simulation runs.

## Discussion

There is increasing interest in applying the more developed models of animal behaviour to plants to explain foraging behaviour. However, important differences between plants and animals exist. For example, animals will often only be able to exploit one ‘patch’ at a time, and thus must decide to exploit that patch or try to find a potentially more rewarding patch. Conversely, roots may simultaneously exploit several patches of varying quality. However, ‘decisions’ are still required by the plant in determining which of these patches to fully exploit (Duke and Caldwell 2000; Hodge 2009). In this work, four different plant types with similar behaviour in isolation were tested under a number of different combinations of conditions of competition and resource distribution. The different plant parameterizations trade growth rate and initial size constraints against the root systems’ effectiveness (*RSE*) in acquiring resources. Even without explicit plastic root responses such as altered root length, root demography etc. (see Hodge 2004, 2006), it is shown that resource distribution could have significant effects on the outcomes of different growth scenarios, with competitive growth being significantly influenced by resource heterogeneity.

The variability in the simulation results arises principally through the environment (resources) and probabilistic nutrient acquisition, and indirectly by the neighbourhood (competitors). The plants possessed no ability to respond directly to their environmental conditions, and therefore ‘grew’ in a purely passive manner. For two of these plant types (types 1 and 2; the faster growing plant types with lower overall relative root system effectiveness), there was no significant difference in final size irrespective of the presence (or nature) of competition or the resource distribution. In contrast, for the other two plant types (types 3 and 4; the slower growing plant types with higher root system effectiveness) there was a markedly different performance depending on growing conditions. The notion of plants of differing growth strategies trading scale against precision of response to a patchy environment is not new (Campbell *et al*. 1991), though is also far from being universally accepted as being the norm across all plant species (see Kembel and Cahill 2005; Kembel *et al*. 2008). However, precision of foraging is not a fixed trait (Wijesinghe *et al*. 2001) and the response by the plant can vary depending on the way nutrient patches are presented to the plant, again highlighting the importance of the attributes of the patch to the response observed.

The presence of competitors can influence root placement and foraging capability (see Jumpponen *et al*. 2002; Cahill *et al*. 2010; Mommer *et al*. 2012), and the outcomes of competitive interactions are not always predictable from extrapolations from growth as monocultures (see Hodge 2003; Cahill *et al*. 2010; Padilla *et al*. 2013), nor in different ecosystems (cf. Jacob *et al*. 2013 with Mommer *et al*. 2010). However, in the model presented here, space and resources per plant were consistent between the different numerical simulations. Thus, the introduction of competition within this framework does not lead to a decrease in available space or resources per plant (which is recognized as an important consideration, although the impact of ‘space’ can be highly variable among species; see McConnaughay and Bazzaz 1991; Murphy *et al*. 2013; McNickle and Brown 2014). It should be noted that the possibility of local overcrowding does result in direct competition between neighbours for locally available resources. Growth into an area of overlap with a competitor will statistically mean growth into an area with lower average resources, reducing the scope for subsequent growth. Plants have been observed to demonstrate root segregation (Schenk *et al*. 1999) which makes sense from this perspective; however in different contexts they have been found to actively proliferate into areas of competition (Hodge *et al*. 1999*c*; Robinson *et al*. 1999). In patchy heterogeneous conditions, the increase in average performance by Plant types 3 and 4 when grown as monocultures as opposed to in isolation highlights an increased ability to exploit available resources. At the population level, both plant types had better per plant performance than when grown in isolation, reflecting the acquisition of a higher proportion of the available resources on average.

Although Plant types 1 and 2 demonstrate a slight reduction in performance when grown in mixed competition in patchy heterogeneous conditions (Fig. 5) compared with when grown as a monoculture (Fig. 4), this reduction is less than the increase in performance experienced by type 3 (as mentioned, type 4 sees little change in performance). This means that while Plant type 3 enjoys an advantage when grown in mixed competition, that advantage is not wholly at the expense of its competitors.

When observing real plants and their performance, behaviour and response to different environmental conditions and stresses, the consistent (if perhaps unhelpful) message is that results are context sensitive (reviewed by Hodge 2004; Karst *et al*. 2012). A remarkable number of different root traits that have been demonstrated to be important for nutrient acquisition from a heterogeneous or ‘patchy’ nutrient environment under different experimental conditions (Hodge 2004; Cahill and McNickle 2011). It follows that any model hoping to capture and replicate all observed behaviour is necessarily going to require a level of complexity and parameterization which, even if it were possible and the required knowledge and understanding were available, would negate the need for such models in the first place. In this work, elements such as plastic responses to the environment are omitted in favour of isolating and investigating mechanistic and stochastic-driven impacts of environmental heterogeneity on growth and competition.

The strength of the work presented here is the use of foraging analogies developed elsewhere to condense a number of these complex traits into two essential mechanistic factors: root system ‘growth’ and ‘effectiveness’ (*RSE*). By categorizing these factors (fast/slow growth, high/low *RSE*) and normalizing so that isolated plants in homogeneous environments behave identically on average, it is possible to isolate the predicted influence of these factors at the individual, population and community level in both homogeneous and patchy environments.

Day *et al*. (2003) observed that populations, when grown under conditions of varying levels of scale and heterogeneity, demonstrated little change in population level yields providing the same total levels of nutrient supply were available. Similarly, Casper and Cahill (1996, 1998) observed soil nutrient heterogeneity had no impact upon productivity or population structure of *Abutilon theophrasti* Medik. monocultures. In contrast, Hutchings and Wijesinghe (2008) observed resource distribution having a distinct effect on overall population level yield. These contrasting results demonstrate the importance of context sensitivity, and the work presented here displays both of these types of behaviour depending on plant characteristics and community composition.

The modelling framework developed here is, to our knowledge, unique in its consideration of stochastic root system growth, maintenance and competition in heterogeneous environments. O'Brien *et al*. (2007) move beyond the traditional ‘zone of interaction models’ (where interaction and overlap between root systems are typically controlled by predefined rules; see for example, Berger *et al*. 2002) to use a game-theoretic spatially explicit model to predict root system distribution of two competing plants. By simplifying resource uptake and depletion, the authors are able to solve deterministic equations for optimal (in cost-benefit terms) growth in competition. This reveals information about root proliferation, overlap and below-ground resource foraging consistent with some empirical studies, such as the reduction of lateral root spread in the presence of a competitor, and an increase in lateral root spread with the introduction of resource heterogeneity. However, while the model can accommodate environmental heterogeneity, an essentially deterministic model such as this cannot capture the stochastic growth dynamics present in reality.

Useful comparisons can also be made with Craine *et al*. (2005) and Craine (2006), where continuous-time uptake and growth mechanisms are employed to model root growth and competition, using a spatially explicit set of 2D (horizontal and vertical) root growth simulations and grid-based diffusion at small (cm) scales. This allows inferences to be made about optimal resource allocation and competition, contingent upon these simplifying assumptions, but does not allow generalization to more than two competitors. The modelling framework developed in our work adds a spatially explicit account of stochastic interaction and depletion of patchy resources, and includes multiple individuals and growth strategies; future hybrids of these modelling approaches may prove fruitful in resolving the mechanisms behind the context-dependent empirical results highlighted above.

## Conclusions

This work shows that ideas, and mathematical and computational methods, borrowed from animal growth and foraging can be used to help to disambiguate the many context-dependent results observed in studies of plant root growth and plasticity. Combining complex processes into idealized properties of growth and efficiency allows the roles of resource heterogeneity and intra- and inter-specific competition to be disentangled. Returning to the questions presaged in the Introduction section:

1. How does the growth strategy adopted by a single plant impact upon its performance in a monoculture?
  In homogeneous environments, intra-specific competition has little impact on plant performance regardless of growth strategy. However, different growth strategies can lead to greatly different performance when grown in intra-specific competition in heterogeneous environments. In these conditions, sacrificing growth rate for *RSE* conveys a clear advantage to the population, and at the individual level provides a better chance of being ‘lucky’.
2. When plant species are grown in mixed competition for resources, what is the impact on individual, population and community productivity?
  During inter-specific competition, there is very little difference in performance at the individual, population or community scale in homogeneous environments. However, in heterogeneous environments, the slower growing plants with higher *RSE* perform significantly better (on average and in terms of best performing individuals) than the other species, and also better than when grown in monocultures. Only a small part of this increase is at the direct expense of the other species, resulting in a community-level increase in productivity.
3. What is the role of resource heterogeneity in the above questions?
  In answering the first two questions, it is impossible to avoid the effect of resource heterogeneity. The results underline the fact that the effects of growth strategy, competition and resource distribution on individuals, populations and communities are intrinsically interlinked.

This work highlights the utility of mathematical and computational models to frame complex problems in a relatively simple and tractable form. Within this work, all four plant types operate within the same framework; they differ only in the parameterization of growth and *RSE*. Yet they are shown to display near identical or markedly different behaviour depending on the context. Different aspects of this context can be individually and independently adjusted to isolate the effects of one factor or another. The story which emerges is consistent with the empirical literature; individual factors generally do not have clear impacts on performance. It is only by considering all factors together that the impacts of different factors can be usefully assessed.

In the discussion of these and other experimental results, a large emphasis is placed on context. However, when talking about ‘optimal’ behaviour, and metrics of performance, one has to be mindful of exactly what it means to perform ‘better’ or ‘optimally’ (Currey *et al*. 2007; Preston *et al*. 2010). The results shown here complement experimental evidence in terms of performance and results under a given set of conditions, but a key strength of this approach is that such frameworks can be tested within an evolutionary context (Croft *et al*. 2012). The next step would be to compare the behaviour and performance of different strategies not just over multiple replications, but rather over a series of dependent iterations. It is arguable only when evolutionarily relevant metrics of performance and optimality are considered that a truly relevant context is considered.

## Sources of Funding

This work was funded by the Biotechnology and Biological Sciences Research Council (BBSRC), UK.

## Contributions by the Authors

S.A.C., J.W.P. and A.H. designed the research. S.A.C. performed the research and analysis. S.A.C., J.W.P. and A.H. wrote the paper.

## Conflict of Interest Statement

None declared.

## Supporting Information

The following additional information is available in the online version of this article—

**File S1.** Technical Methodology. Provides a more detailed technical description of methodology, including mathematical and code implementation, and a list of parameters used.

Additional Information
