# Supplementary material for: Fishing for nutrients in heterogeneous landscapes: modelling plant growth trade-offs in monocultures and mixed communities
Source: AoB Plants. 2015 Sep 14;7:plv109. doi: 10.1093/aobpla/plv109 (PMC4641210; doi:10.1093/aobpla/plv109)
Supplement: Additional Information [file supp_7_plv109_index.html]

Fishing for nutrients in heterogeneous landscapes: modelling plant growth trade-offs in monocultures and mixed communities — Fishing for nutrients in heterogeneous landscapes: modelling plant growth trade-offs in monocultures and mixed communities — Additional Information 

# Fishing for nutrients in heterogeneous landscapes: modelling plant growth trade-offs in monocultures and mixed communities

## Additional Information

Additional Information

- Additional Information - Docx file
