# Supplementary material for: Fishing for nutrients in heterogeneous landscapes: modelling plant growth trade-offs in monocultures and mixed communities
Source: AoB Plants. 2015 Sep 14;7:plv109. doi: 10.1093/aobpla/plv109 (PMC4641210; doi:10.1093/aobpla/plv109)
Supplement: Additional Information [file supp_plv109_plv109supp.docx]

# Technical Methodology

The following material describes the methods process, including parameters and equations which govern the initialisation of the simulations (environment and neighbourhood generation) as well as the running of the simulations themselves (plant growth and resource capture).

Table 1: of parameters and variables utilised within the simulation along with functional description and value (where applicable).

| **Parameter/variable name** | **Function** | **Value** |
| --- | --- | --- |
| *T* | Total time over which each simulation runs | 1 |
| *t* | Current time | - |
| *dt* | Time step length | 10^-4 |
|  |  |  |
| *P* | Number of plants within simulation | 1 (isolation),  64 (competition) |
| *S* | Environment area | P |
| *N* | Number of individual resource points | 100**P* |
| *x* | Performance normalising parameter | 0.785 |
| *p*_tot_ | Total resource quantity within environment | *x*P* |
| *p* | Quality of each individual resource patch | *x*/100 |
|  |  |  |
| *Θ* | Angle of inter-patch walk step for heterogeneous resource distribution | 0-2π |
| *r* | “Inverse mean” parameter in Pareto distribution for heterogeneous resource distribution | 8 |
| *Λ* | “Patchiness” parameter in Pareto distribution for heterogeneous resource distribution | 2.1 |
|  |  |  |
| *L*_max0_ | Initial upper size limit | See figure 2 |
| *L*_max_ | Current upper size limit | - |
| *g* | Growth rate | See figure 2 |
| *f* | Current root system efficiency (RSE) of an individual | - |
| *f*_0_ | Initial RSE | See figure 2 |
| *f*_fin_ | Final RSE | See figure 2 |
| *m* | Marginal benefit factor | 0.5**g* |
| *A* | Size (area) of individual plant | - |
| *R* | Radius of individual plant | - |

**Environment generation.**

*Uniform*: each patch location is sampled independently from a two dimensional U[0,$\sqrt{S}$] distribution. This produces a statistically uniform spatial distribution.

*Heterogeneous*: The first patch location is sampled from a two dimensional U[0,$\sqrt{S}$] distribution. For each subsequent patch, an angle θ is sampled from a U[0,2π] distribution, and a distance sampled from a Pareto type two distribution. This patch is then placed according to this distance and angle from the previous patch (respecting the periodic boundaries of the environment).

The Pareto distribution (specifically, a non-standard Pareto distribution of the second kind; see Johnson *et al.* 1994) is sampled to generate inter-patch distances, *d*, with the probability density function given by

$f\left( d \right)=\frac{r\alpha^{r}}{{(\alpha+d)}^{r+1}}, (d>0)$.

Reparameterising according to

$$\alpha=\frac{r-1}{\lambda}$$

allows for two parameters, λ and *r*, which independently define the mean distance between patches and the heterogeneity of the overall distribution, respectively, giving control over levels of “patchiness” in the resource distribution (see, for example, James *et al.* 2005).

A value of *r*=2.1 provides a high level of heterogeneity (or “patchiness”) whilst maintaining a finite variance in distribution, and a value of $\lambda$=8 is chosen by experiment to provide a good coverage across the environment (i.e. allows for patches to be distributed across the entire range of the environment).

**Plant placement**

For plants grown in isolation, the plant is simply placed at the centre of the generated environment (i.e. coordinates [$\sqrt{S}/2$],$\sqrt{S}/2$). Due to the construction of the environments (in terms of resource distribution and periodic boundaries), there is no bias effect on the plant from placing it in this manner.

For plants grown in competition, each plant is placed independently according to sampling from a two dimensional U[0,$\sqrt{S}$] distribution. This results in a statistically uniform spatial distribution of plants within the environment. This random process naturally allows for the possibility of aggregation/crowding of neighbouring plants.

**Plant growth**

The simulation runs, and the plants grow, whilst time, *t*, is less than the total simulation time, *T*. Whilst this is the case, time increments by a fixed time step *dt*. At each time step, growth, root system efficiency (*f)* decline (where applicable) and potential resource capture occur. Plant growth can be broken down into two steps:

*Deterministic growth*

Deterministic (i.e. non-resource dependent) growth by area is simply given by

$A\left( t+dt \right)=A(t)+g*dt$.

It follows that the corresponding radial growth from the centre of the plant is given by

$$R\left( t+dt \right)=\left( \frac{A(t)+g*dt}{\pi} \right)^{1/2}.$$

Plants keep growing until their area, *A*, is equal to their upper size limit, L_max_. At this point, deterministic growth ceases, and subsequently no further resources can be encountered.

The changes to *f*, the root system efficiency, are governed by the following equation:

$f\left( t+dt \right)=f\left( t \right)-(f_{0}-f_{fin})/(L_{max0}/(g*dt))$.

The value of *f* keeps reducing until it reaches its final lower limit, *f*_fin_. It follows that if the root system efficiency is constant (i.e. f_fin_ is equal to f_0_), then this equation simply reduces to *f*(*t*+*dt*)=*f*(*t*), and no change occurs.

*Resource acquisition and stochastic growth*

Whenever the area occupied by a plant overlaps an available resource patch, the plant is potentially able to acquire this patch. At each time step, available resources within the plant’s area are checked, and acquisition is governed by the probability defined by the plant’s root system efficiency value, *f*. Individual patches successfully captured by the plant are removed from the environment, and the resources are used by the capturing plant for growth.

Stochastic growth is determined by the successful capture of available resources from the environment. Upon successfully capturing an available resource patch, the plant experiences an instantaneous growth (i.e. a jump in size) equal to the product of the marginal benefit of the patch (*p*) and the marginal benefit factor, *m*, of the plant, i.e.

$$A^{'}=A+p*m.$$

As well as the plant receiving this instantaneous growth, the upper size limit, *L*_max_ , is also increased by the same amount, i.e.

$L\_\max'=L\_\max+p*m$.

In this way, the plant’s size under resource limited growth is controlled both in terms of growth rate, and achieved size.

The simulation ceases at time *t=T*. At this point, all plants’ root system efficiency values will be equal to their final value, f_0,_ and each plant will be equal in size to their upper limit, L_max._

James A, Baxter P, Pitchford J (2005) Modelling predation as a capped rate stochastic process, with applications to fish recruitment. J R Soc Interface 2:477-487

Johnson NL, Kotz S, Balakrishnan N (1994) Continuous univariate distributions-1, Second Edition. New York: Wiley-Interscience, pp 574-575

**Sample MATLAB Code**

The following provides code samples demonstrating how the model is implemented and run. This example demonstrates the code for a lone plant in isolation.

The first step is to initialise the model with the relevant parameters, from which the RSE increment is calculated.

clear all

T=1; %total time

dt=10^-4; %time step

P=1; %no. of plants

S=P; %environment area

s=sqrt(S); %environment length

N=100*S; %number of resource points

r = 8; %"inverse mean" Pareto parameter

lam = 2.1; %"patchiness" Pareto parameter (>2)

x=0.7850; %normalising parameter

p_tot=x*s; %total resource quality

p=p_tot/n; %individual point quality

L_max0=0.2; %initial upper size limit

g=0.2; %growth rate

m=0.5*g;

f_0_type=0.5; %initial RSE

f_fin_type=0; %final RSE

f_incr=(f_0-f_fin)/(L_max0/(g*dt)); %RSE time step increment

Next the environment is populated with resource patches and plants. The resource patch distribution depends on the environment type (i.e. homogeneous or heterogeneous; the correct code sample needs to be chosen accordingly). The distances from each patch to the plant are then calculated.

P_loc=[s/2,s/2]; %plant coordinates

%UNIFORM PATCH DISTRIBUTION

pat_loc=s*rand(N,2); %patch coordinates

%

%HETEROGENEOUS PATCH DISTRIBUTION

pat_loc=zeros(N,2); %preallocate

pat_loc(1,:)=s*rand(1,2); %first patch coordinates

for i=2:N

u_ang=2*pi*rand; %sample angle

temp=rand; %sample for step length calc

%step length

u_dist=((lam-1)/r)*((1-(1-temp)^(1/lam))/(1-temp)^(1/lam));

%patch coordinates

pat_loc(i,:)=mod(patches_loc(i-1,:)+u_dist*[cos(u_ang),sin(u_ang)],s);

end

%

%patch distances from plant

pat_loc_rel=(P_loc(1)-pat_loc(:,1)).^2+(P_loc(2)-pat_loc(:,2)).^2;

The model is then set to run, and does so within a ‘while’ loop whilst *t*<*T* (i.e. until the

full time limit is reached). At each time step, the time is incremented and

deterministic elements (growth and RSE decline) implemented. The model then

checks for available resources, and performs a random sample to determine

successful patch acquisition. The growth benefits from any successfully acquired

patches are then applied.

t=t+dt; %increment time

A=A+g*dt; %plant area growth

R=sqrt(A/pi); %plant radius

f=f-f_incr; %increment RSE

pat_check=pat_loc_rel-R^2<0; %find patches within plant area

u=rand(size(pat_loce_rel,1),1); %random sample for acquisition

u=(u<f); %sample check

pat_acq=pat_check.*u==1; %acquired patches

pat_loc_rel(pat_acq)=[]; %remove acquired patches from environment

A=A+numel(find(pat_acq))*p*m; %plant area growth due to patch acquisition

R=sqrt(A/pi); %plant radius

This concludes at time *t*=*T*.

The code to run for multiple plants is simply modified by adding an extra dimension

to relevant vectors/matrices for each plant. At each time step a random order is

chosen to update growth and acquisition for each plant. When a resource point is

successfully acquired by a plant, it is removed from the environment for all plants to

avoid multiple captures.

Full versions of the code to reproduce the data behind the figures are available on

request to the authors.
